# Evaluation of Dry Electrodes in Canine Heart Rate Monitoring

**DOI:** 10.3390/s18061757

**Published:** 2018-05-30

**Authors:** Juhani Virtanen, Sanni Somppi, Heini Törnqvist, Vala Jeyhani, Patrique Fiedler, Yulia Gizatdinova, Päivi Majaranta, Heli Väätäjä, Anna Valldeoriola Cardó, Jukka Lekkala, Sampo Tuukkanen, Veikko Surakka, Outi Vainio, Antti Vehkaoja

**Affiliations:** 1 BioMediTech Institute and Faculty of Biomedical Sciences and Engineering, Tampere University of Technology (TUT), P.O. Box 692, FI-33101 Tampere, Finland; vala.jeyhani@tut.fi (V.J.); jukka.lekkala@tut.fi (J.L.); sampo.tuukkanen@tut.fi (S.T.); antti.vehkaoja@tut.fi (A.V.); 2 Department of Equine and Small Animal Medicine, University of Helsinki (UH), P.O. Box 57 (Viikintie 49), FI-00014 Helsinki, Finland; sanni.somppi@helsinki.fi (S.S.); heini.tornqvist@helsinki.fi (H.T.); anna.valldeoriola@helsinki.fi (A.V.C.); outi.vainio@helsinki.fi (O.V.); 3 Institute of Biomedical Engineering and Informatics, Technische Universität Ilmenau, Gustav-Kirchhoff-Straße 2, 98693 Ilmenau, Germany; patrique.fiedler@tu-ilmenau.de; 4 Research Group for Emotions, Sociality, and Computing, Faculty of Communication Sciences, University of Tampere (UTA), Pinni B Building, Kanslerinrinne 1, FI-33014 Tampere, Finland; julia.kuosmanen@uta.fi (Y.G.); paivi.majaranta@uta.fi (P.M.); heli.vaataja@tut.fi (H.V.); veikko.surakka@uta.fi (V.S.)

**Keywords:** dry electrode, heart rate canine

## Abstract

The functionality of three dry electrocardiogram electrode constructions was evaluated by measuring canine heart rate during four different behaviors: Standing, sitting, lying and walking. The testing was repeated (n = 9) in each of the 36 scenarios with three dogs. Two of the electrodes were constructed with spring-loaded test pins while the third electrode was a molded polymer electrode with Ag/AgCl coating. During the measurement, a specifically designed harness was used to attach the electrodes to the dogs. The performance of the electrodes was evaluated and compared in terms of heartbeat detection coverage. The effect on the respective heart rate coverage was studied by computing the heart rate coverage from the measured electrocardiogram signal using a pattern-matching algorithm to extract the R-peaks and further the beat-to-beat heart rate. The results show that the overall coverage ratios regarding the electrodes varied between 45–95% in four different activity modes. The lowest coverage was for lying and walking and the highest was for standing and sitting.

## 1. Introduction

Animal computer interaction (ACI) is a new, emerging discipline with significant commercial potential [1]. Its roots are in agriculture, animal welfare, and animal behavior research [2]. However, research specifically focusing on interaction between humans, animals and technology is still rather scarce, and mostly exploratory [3]. At the same time, especially the business related to tracking and monitoring of pets’ behavior is growing fast [4]. One emerging direction in developing measurement technology is to interpret and understand how animal well-being relates to dog’s emotions and emotional problems [5]. Dogs can suffer from anxiety, phobias and aggression. A range of serious anxiety-related behavior problems, including noise phobias, separation anxiety and aggression have been found to exist in dogs [6].

It has been suggested that measurement of heart rate (HR) and heart rate variability (HRV) could reflect a dog’s emotional responses and stress, as well as serve as an indicator of a dog’s welfare [3,5]. However, there is a need for further validation on the association of a dog’s well-being with HRV [6,7,8]. In addition to that, the measurement technology for obtaining HRV information should be improved to suit for continuous everyday use. Polar technology [9], for example, has been validated for HRV measurement in dogs. However, the studies have applied relatively narrow circumstances in which the dog was steady in a standing position [10,11]. Thus, there is a clear need to develop technology that could function reliably in more versatile conditions, like standing and walking outside of clinical contexts and other similar situations.

Due to the above reasons, there has been a growing interest to monitor pet HR and respiratory activity. Electrocardiogram (ECG) electrodes have also been reported to be used with animals [8]. Typically, the HR of dogs has been measured using rubber electrodes (e.g., Polar^®^ chest strap, Polar Electro oy, Kempele, Finland) with electrically conductive gel [12] or adhesive disposable electrodes that require shaving of the fur [5]. These techniques are questionable when HR is monitored outside the clinical applications in everyday environments. Recurrent shaving of a dog’s fur is not convenient for the dog or the owner, nor is the use of conductive gel. Despite the conductive gel, poor electrode conduction may cause problems while the dog is moving freely [12]. This mandates a need for a maintenance-free electrode system which could provide the HR data constantly and reliably for long periods of time.

In this study, the main focus was to concentrate on the dry electrodes that could be used with thick and dense haired animals without shaving the fur and also without using electrolyte gels or other conductivity promoters. Our goal was to find out whether these electrodes could be used in maintenance-free canine heart rate monitoring. A literature review revealed that different types of pin electrodes, patches and tape electrodes have been used in animal HR measurements. In this study, the pin type electrode was taken into a more detailed investigation as it is the type of electrode that demonstrated promising results when placed on the hairy regions of a dog (e.g., [13]). Two new types of pin electrodes and one new molded polymer electrode were created and tested in this study. Preliminary measurements were carried out to obtain information about the reliability and usefulness of the electrodes in different use scenarios, and produce insights into further investigation and improvement of the measurement technology. The electrodes were evaluated in terms of heart rate coverage. The found individual heartbeats were further used to calculate heart rate coverage during different activities. In particular, the effect of different dog activity modes—standing, sitting, lying and walking—to the heart rate coverage was in the study focus. The results were verified by a human observer to ensure that the algorithm performed correctly.

## 2. Materials and Methods

The measurement system consisted of four electrodes in their respective housings. These were attached to a harness, which the dog wore during the measurements. The data was collected as an ECG potential measurement with a portable device, which along with the ECG data also recorded the accelerometer information. This collected data was later downloaded from the measurement device and further processed offline.

### 2.1. The Mechanical Setup of the Electrodes

Electrodes and the housings in which the electrodes were attached during the measurements were designed and constructed. Additionally, a wearable neoprene harness was designed and fabricated for fixing and attaching the electrodes to the desired locations on the dog’s body. At first, fixtures and fixture molds were designed, CAD modeled and 3D-printed from polylactide (PLA) using an FDM printer (Prusa i3 MK2, Prusa Research s.r.o., Prague, Czech Republic). Later, silicone rubber fixtures were cast into the 3D-printed molds.

**Figure 1:**
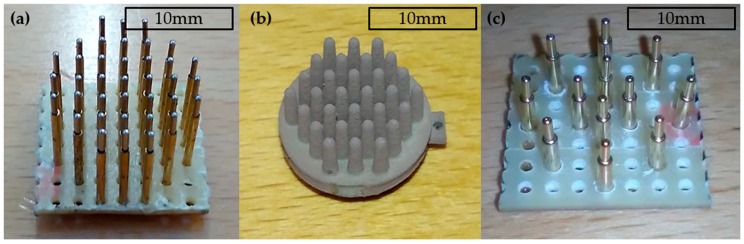
Electrodes: (**a**) The spring-loaded j75-1 test pins; (**b**) The Ag/AgCl-coated polymer electrode; (**c**) The electrode with gold-plated spring-loaded test pins.

Two different spring-loaded electrodes were newly constructed and evaluated. Additionally, polymer electrodes with Ag/AgCl coating, prepared by the University of Ilmenau, were studied [14]. The spring-loaded electrodes were fabricated by soldering spring-loaded test pins in an array form on a printed wiring board. The first electrode type contained 37 test pins of type j75-1. Additionally, this electrode had a 5 mm thick PLA plate on it, as shown in Figure 1a. The second electrode type, a non-spring-loaded Ag/AgCl-coated polymer electrode with 30 pins, is shown in Figure 1b and the third electrode with 12 gold-plated test pins of type Tangda M1071, is shown in Figure 1c. The height of the pins of all electrodes was approximately 6 mm. All the pins in the electrodes were electrically connected together and, therefore the electrical potential was integrated over the contact area of all pins. The electrode configurations are also listed in Table 1.

### 2.2. The Housing of the Electrodes

**Figure 2:**
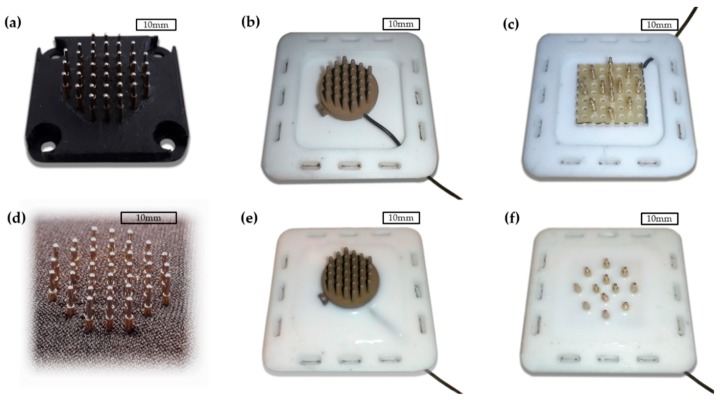
The electrodes embedded into housings: (**a**) Peak electrode with PLA plate; (**b**) Ag/AgCl-coated polymer electrode before the second casting; (**c**) Gold electrode before the second casting; (**d**) Peak electrode in the neoprene fixture; (**e**) Ag/AgCl coated polymer electrode in the silicone rubber fixture; (**f**) Gold electrode in the silicone rubber fixture.

Two different electrode housings were fabricated. The first housing was constructed of a printed PLA plate, shown in Figure 2a, and a sheet 2 mm thick, textile-coated neoprene rubber through which the spring-loaded pins were pushed, as Figure 2d illustrates.

**Figure 3:**
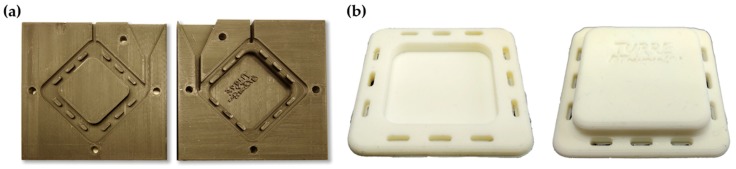
(**a**) Mold for the silicone rubber housing casting; (**b**) Cast silicone rubber electrode housing.

The second type of housing was cast from silicone rubber (Zhermack ZA RTV 30-60, Zhermack SpA, Badia Polesine, Italy) into a custom designed 3D-printed two-part casting mold. This housing was used with both polymer and gold electrodes. Figure 3a shows a top and bottom view of the silicone rubber mold. The molded fixation parts are shown in Figure 3b. The aim of using silicone rubber in the housing was to enable more evenly distributed surface pressure and more comfortable wearing of the electrode.

After the initial casting, the electrodes were placed in to the cast silicone fixtures and a second casting was performed in order to make an integral electrode silicone rubber structure. This is illustrated in Figure 2b,c, where the polymer and gold electrodes are placed in to the initial castings, respectively. The polymer and gold electrodes after the second silicone rubber casting are shown in Figure 2e,f, respectively.

### 2.3. The Harnesses

**Figure 4:**
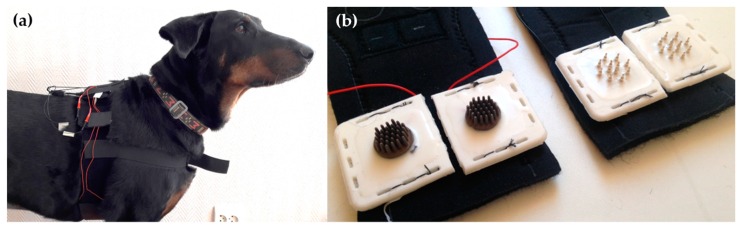
(**a**) The second version of the neoprene harness placed on the dog; (**b**) Polymer and gold electrodes attached to the neoprene harness.

Two different harnesses were used during the measurement to ensure a proper location of the electrodes and as equal surface pressure conditions as possible for all electrodes. Both harnesses were constructed of textile-coated elastic neoprene rubber. The first version was made of 2 mm neoprene with a velcro-coated outside layer. The second version was made of 3 mm thick neoprene and in addition to the velcro layer, it had polyester lining. The harnesses consisted of separately adjustable parts (chest belt around the dog’s thorax and frontal belt around the chest), which were attached together with velcro fastener ensuring that the harnesses were ergonomic and comfortable for dogs. The electrodes were integrated to the lower part of the belt around the chest, two electrodes near to both armpits. The harnesses with the integrated electrodes and monitoring device were put on the dog as shown in Figure 4a. Four electrodes were used in a single measurement setup the of locations of the particular electrodes. The attachment of the polymer and gold electrodes can be seen in Figure 4b. The hair of the dogs was not shaved for the measurements. Neither was any electrode paste or other electrolyte used.

### 2.4. Measurement Electronics

A custom made physiological monitoring device named SpiritCor9D was used to record ECG data with 250 Hz sampling frequency. In particular, the measurement device recorded three channels of ECG, impedance pneumography signal, and 3D acceleration and gyroscope data. The data was stored on the internal memory of the monitoring device and later extracted for the analysis performed on a PC computer. Unshielded electrode wires were used to connect the measurement electronics with the electrodes.

### 2.5. Measurement Trial Configuration

The animal experiments were conducted at the University of Helsinki. The procedures were approved by the Ethical Committee for the Use of Animals in experiments at the University of Helsinki (statement 2/2018). Three dogs were invited to participate in the test measurements. The dogs were two female Beauce Shepherds (Dog 1: 9 years, 32 kg; Dog 3: 8 years, 38 kg, short hair with undercoat) and one male Hovawart (Dog 2: 11 years, 35 kg, and long hair with undercoat).

The testing was organized in 36 different measurement scenarios (three electrodes, three dogs, four activities). Each of these measurement scenarios were repeated nine times (n = 9) in three testing sessions to obtain more reliable measurement results. One testing session consisted of three cycles of a sequence of behaviors, namely, standing 60 s; sitting 60 s; lying 60 s; and walking 60 s, with a settling time of approximately 10 s. The blocks were performed in succession forming a total duration of 12 min per session. The four different activity or posture modes were: standing, sitting, lying and walking. During the walking mode, the handler moved at walking speed while the dog followed unleashed keeping the same speed. The dogs were allowed to trot and pace to keep their speed. Walking mode included multiple sharp turns. Before each testing session, the dog was freely moving for one minute, to get accustomed to the harness. The measurement harness was taken off and put back on between the measurement sessions.

### 2.6. Heartbeat Detection and Heart Rate Analysis

The three electrode types were compared using ECG R-peak coverage ratios. R-peak was defined as the ratio between a successfully recovered R-peak time divided by the total test measurement time. Thus, a fully recovered HR data would yield a coverage ratio of 1.

R-peak detection is a standard operation in HR monitoring devices. There is a large variety of methods developed and published for R-peak detection from the measured EGC data, some of them were developed especially for integration on a low-performance microcontroller [15,16,17]. A pattern matching type R-peak detection method, derived from the method proposed by Dobbs et al. [16] was applied to detect the R-peaks in this study. The principles of the algorithm used in this evaluation have been presented with ECG compression algorithms [18]. In short, the data firstly filtered and scaled, and after this a pattern matching method was applied as follows.

**Figure 5:**
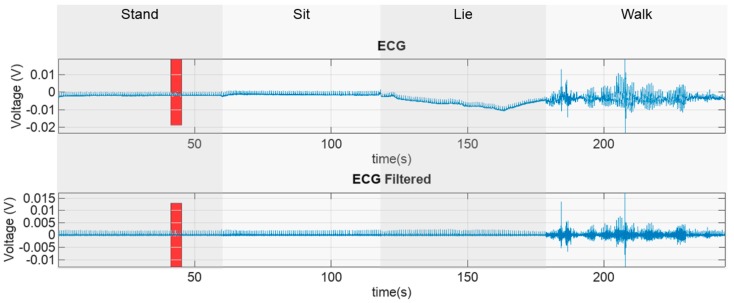
Illustration of the measured and filtered ECG data. Red rectangles denote the parts of the original signal that will be depicted in detail in Figure 6.

**Figure 6:**
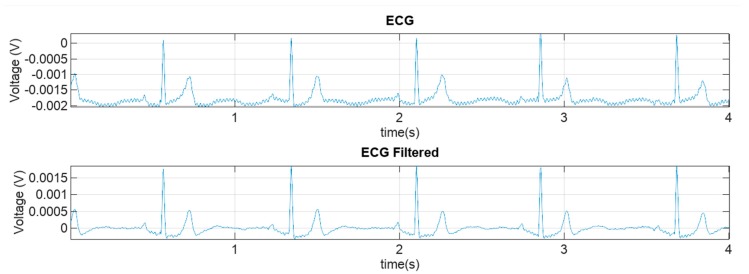
Zoomed-in raw and filtered ECG data. The data comes from the original signals presented in Figure 5 that are marked by red rectangles.

Coverage ratios were computed for each activity mode and each dog. The measured raw ECG and filtered ECG data with different respective activity modes, is presented in Figure 5. The measurement data was manually split into to groups. In the figure, the horizontal time axis is matched such that the graphs are vertically aligned. The red rectangle in each of the graphs represents the area that is zoomed in to reveal more details in the data. The zoomed graphs (red rectangles from Figure 5) that represent a time period of 4 s, are shown in Figure 6.

**Figure 7:**
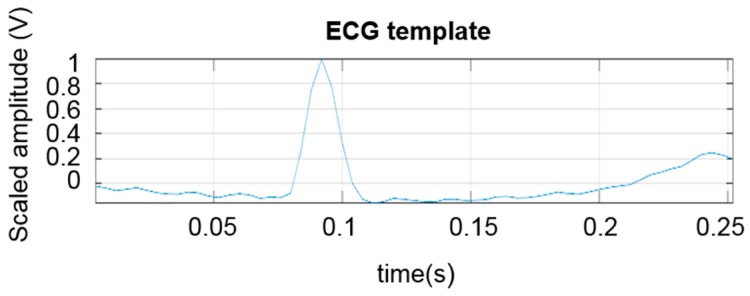
A typical scaled EGC template pattern that was used in pattern matching.

The processing was carried out by first applying a fourth order 2–30 Hz Butterworth forward-backward band pass filtering. Next, a pattern finder procedure was applied to the filtered data to find individual heartbeats in the ECG and to obtain the HR. In this procedure, first a typical template (Figure 7) of ECG pattern is selected. In particular, the pattern was obtained by visually analyzing the filtered ECG data and selecting a typical ECG pattern. The template pattern contains all essential components of an ECG from P to T waves in order to minimize the false detection of the complexes. This templated pattern was 250 ms in length in all analyzed computations. The template length was selected empirically to contain all the ECG components of one cardiac cycle from atrial depolarization (P-wave) to ventricular repolarization (T-wave). Next, the template was normalized such that the maximum value of the template (R-peak) corresponded to a value of 1 and all other values in this ECG vector were scaled linearly with the same normalizing factor.

After the template assignment, the data was split into windows to perform adaptive amplitude scaling for the data. This processing phase started by defining an observation window length. The length was selected in such a way that it was longer than the maximum normal R-R-interval to ensure that in this window at least one R-peak would be present. In this particular case, the window length was selected to be 2 s to ensure the presence of the potential R-peak. Next, the data in the observation window was processed such that the sample with a maximum value in the data window was scaled to 1 and all other values in the vector were scaled linearly with the same coefficient. The actual pattern matching was then performed by computing the sum of the squared difference of the template and the observation window when the template was moved along the observation window vector. This windowing procedure was repeated to process the whole data.

In order to distinguish the matched pattern, a threshold of the distances which identifies a matched pattern was defined. Below this threshold level, the two vectors were interpreted to match and thus an R-peak was detected. The threshold level was visually observed from the output of the pattern match distance calculation. Care was taken to select the practical threshold level empirically such that false R-peak detections were minimized. In this particular study, an empirically defined threshold value of 0.3 was used. This yielded to less than 1% of false R-peak detections when the result was visually inspected against the filtered ECG data.

**Figure 8:**
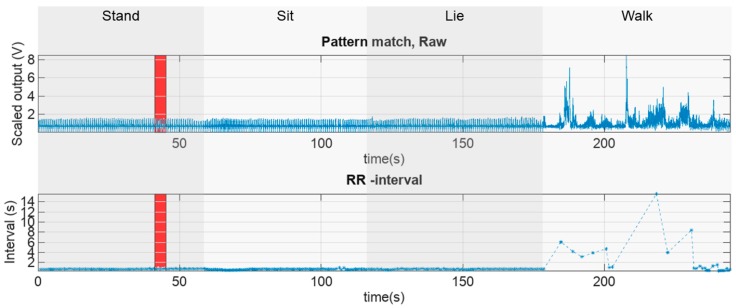
Raw pattern match data and its respective RR interval graph. Red rectangles denote the parts of the original signal that will be depicted in detail in Figure 9.

**Figure 9:**
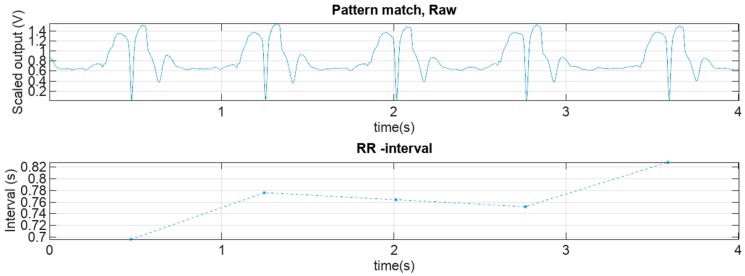
A zoomed-in representation of the scaled vector distance from the template pattern and the respective zoomed-in RR interval graph.

Thresholding the pattern matcher output data gives the location of the pattern match. This can also be interpreted as the location of the R-peak or the local minimum of the template data distance. This data was then used to compute the R-R interval signal values, which is the time elapsed between two detected R-peaks. A graph of the raw pattern match data and the respective RR-interval tachogram along with the different respective activity modes, is presented in Figure 8. A zoomed in (red rectangle) representation of these values can be seen in Figure 9. Finally, the coverage was computed as the ratio of successfully detected R-R interval signal time versus total measurement time.

## 3. Measurement Results

Electrode coverage ratio averages (n = 27/activity) including all dogs and all electrodes varied between 0.45 and 0.95 with respect to the different activity. The standard deviation of the RR-interval coverage varied between 0.08 and 0.36. Furthermore, the median values varied between 0.44 and 0.99. Finally, the coverage ratio ranges varied between and 0.35 and 0.98. These average, standard deviation, median and range values are listed in Table 2. When additionally considering all the measurement scenarios and activity modes in separate cases regarding different electrodes (n = 9/activity), the average coverage ratios varied between 0.18 and 0.96, standard deviations between 0.06 and 0.42, medians between 0.17 and 1.00, and finally ranges between 0.08 and 0.97. The results are listed in Table 3 according to each electrode. The maximum and minimum values in all categories are highlighted in a bold typeface in Table 2 and Table 3.

**Figure 10:**
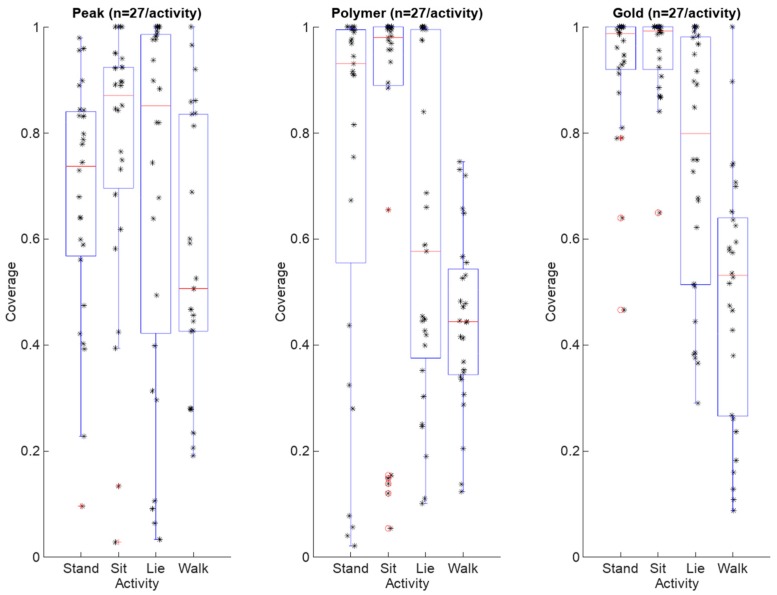
Combined heart rate coverage boxplots with observation points which represent a coverage ratio computed from a 60 s measurement case. The observations are marked with stars while the outliers are marked with red circles.

**Figure 11:**
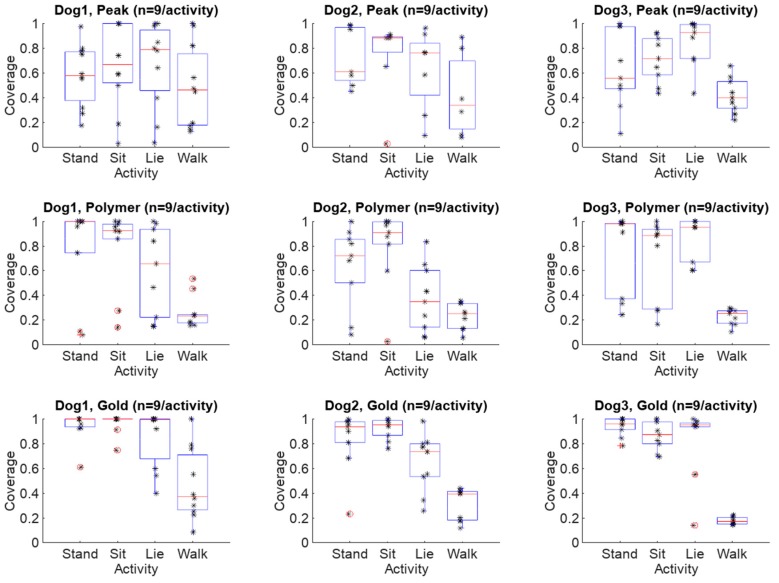
Boxplots of coverage ratios in different configurations with the respective observation points included in the plots. The observations are marked with stars while the outliers are marked with red circles.

The heart rate/heartbeat detection coverages for each electrode type and activity obtained with all three dogs and in all test repetitions (n = 27/activity) are presented as boxplots in Figure 10. In Figure 11, the same results are presented for each dog separately (n = 9/activity).

Statistically significant differences in electrodes were investigated by performing a Mann-Whitney test on the coverage data. The data was organized such that the electrode data was accumulated (n = 27 for each activity mode) to represent each electrode in each activity mode. The computed *p*-values for the null hypothesis test are listed in the Table 4.

The analysis shows that there are statistically significant differences in the electrode coverage performance especially with standing and sitting activity modes. In this analysis null hypothesis is rejected when *p* < 0.05 and these cases are marked with bold typeface in the Table 4. The null hypothesis is rejected when there is a statistically significant difference detected between the test pairs. Therefore, in the standing and sitting activity modes peak electrodes are separable from both polymer and gold electrodes.

## 4. Discussion

In this study, we evaluated the performance of three different dry electrodes for maintenance-free canine heart rate monitoring. The results showed that the electrodes performed differently in certain activity modes when evaluated in terms of heart rate coverage ratios. The highest coverage ratios (over 90%) were achieved with relatively stationary postures when the dogs were standing or sitting; while the lying and walking modes resulted in coverage ratios of 75% and 49% on average, respectively. The results are in line with those obtained by Brugarolas [13].

There are several factors that might have affected the performance of the electrodes. Likely, the variation in the coverage ratios may be due to the thickness and quality of the animal hair as well as the flexibility and elasticity of the electrodes. Additionally, the actual electrode length, pin density (pin-to-pin distance), and effective contact pressure may have contributed to the performance by affecting the penetration of the electrode through the hair. Contact pressure was shown prior to considerably affect the contact stability and the signal quality of dry-contact electrodes.

Beyond the different activity modes, there are other interfering factors due to the physiological properties of the tested dog breed. Further, it appears that there are also anatomical factors like the structure of the thorax (or even torso) area, which can have an effect on how the electrodes are able to retain the necessary skin contact. The dog thorax shape changes in different postures, affecting the position and the tension of the electrodes in the harness. The effect of the thorax shape change on the coverage ratio is probably the best observed in lying position, where the thorax flattens when compared to, for example, standing position. Future long-term studies are needed to investigate and improve the dry electrode design to adapt with canine thorax shape changes.

The limitations of this study are related to the relatively small sample size of the dogs and dog breeds. Therefore, even though the results certainly reveal logical behavior of the electrode harness combination in the different activity modes, it is possible that the results of this study are not generalizable as such. Even though the data was carefully visually inspected to ensure the reliability of the R-peak detection, some part of the uncertainty in the measurement results is also possible because of the lack of a validated ECG measurement reference (e.g., Holter device). However, it is estimated that this does not contribute significantly to the measurement results reliability.

This is an ongoing work and we presented the initial results of the first trials of canine ECG electrode measurements, showing that the proposed electrodes can be used to record ECG with sufficient quality for further processing into reliable HR data. When thinking of the mass production of the electrodes studied, it can be concluded that all the tested electrode structures are relatively simple constructions and conceptually mass producible. Whereas, the spring-loaded electrodes are a multipart construction, the polymer electrode could be a single shot injection molded part. This makes the polymer electrodes potentially less expensive to produce in large production volumes.

In future studies, other measures could also be used, such as simultaneously measured cortisol in saliva and behavior, to improve the interpretation of heart rate data with regard, for example, to a dog’s emotional responses and stress [19]. Also, several topics may be addressed in the future research such as improving the accuracy and the reliability of the proposed R-peak detection method by applying adaptive methods, multiple pattern recognition and dynamic thresholding.

## 5. Conclusions

In this work, three types of dry ECG electrodes were studied. The evaluation included studying the effect of different activity modes in the electrode evaluation. In particular, the interest was in the dry electrodes that could be conveniently used in the canine ECG measurement without the need of gel or shaving of the animal hair. To carry out the evaluation, a method for QRS complex and R-R interval from the ECG signal was constructed. The R-R interval coverage ratio as an output measure of the performance of the electrodes was evaluated to be as high as over 0.9 in favorable measurement scenarios. However, there was rather large variation in the computed coverage ratios in different cases, which may suggest that the studied electrodes may not be as suitable for all dog breeds. It was found that the metal spring-loaded type electrodes work rather satisfactorily with short-haired breeds while the longer and thick-haired dogs may be more challenging for this particular electrode type. Heart rate monitoring in more dynamic activity modes such as walking was found to be less reliable in terms of heart rate coverage.

## Figures and Tables

**Table 1:** Electrode housing configurations used in the study.

Electrode	Peak	Polymer	Gold
Contacts	j75-1	Ag/AgCl-polymer	M1071
Housing	Neoprene	Silicone rubber	Silicone rubber
Pins	37	30	12

**Table 2:** Average coverage ratios and their respective standard deviations of each electrode in all activity modes combined. The maximum and minimum values are highlighted in bold typeface.

Activity	Stand				Sit				Lie				Walk			
Electrode	Avg	Sd	Md	R	Avg	Sd	Md	R	Avg	Sd	Md	R	Avg	Sd	Md	R
Peak	0.68	0.23	0.74	0.88	0.76	0.26	0.87	0.97	0.70	0.35	0.85	0.97	0.57	0.26	0.51	0.81
Polymer	0.74	**0.36**	0.93	**0.98**	0.81	0.34	0.98	0.95	0.61	0.32	0.58	0.90	**0.45**	0.17	0.44	0.62
Gold	0.93	0.13	**0.99**	0.53	**0.95**	**0.08**	**0.99**	**0.35**	0.75	0.25	0.80	0.71	0.49	0.24	0.53	0.91

**Table 3:** Average coverage ratios with different measurement scenarios regarding the different electrode design. The maximum and minimum values are highlighted in bold typeface.

**Peak**																
**Activity**	**Stand**				**Sit**				**Lie**				**Walk**			
	**Avg**	**Sd**	**Md**	**R**	**Avg**	**Sd**	**Md**	**R**	**Avg**	**Sd**	**Md**	**R**	**Avg**	**Sd**	**Md**	**R**
Dog 1	0.58	0.26	0.58	0.80	0.66	**0.35**	0.67	**0.97**	0.66	0.35	0.79	**0.97**	0.49	0.34	0.46	0.87
Dog 2	0.72	0.24	0.61	0.54	0.74	0.33	0.88	0.89	0.62	0.33	0.76	0.87	**0.42**	0.35	0.34	0.81
Dog 3	0.63	0.31	0.56	0.89	0.71	0.19	0.72	0.49	**0.85**	0.19	**0.93**	0.57	**0.42**	0.15	0.40	0.44
**Polymer**																
**Activity**	**Stand**				**Sit**				**Lie**				**Walk**			
	**Avg**	**Sd**	**Md**	**R**	**Avg**	**Sd**	**Md**	**R**	**Avg**	**Sd**	**Md**	**R**	**Avg**	**Sd**	**Md**	**R**
Dog 1	0.77	**0.39**	**1.00**	0.92	0.78	0.33	0.92	0.86	0.60	0.36	0.66	0.85	0.27	0.14	**0.23**	0.38
Dog 2	0.63	0.33	0.72	0.92	0.80	0.32	0.91	**0.97**	0.37	0.28	0.35	0.77	**0.23**	0.11	0.25	0.30
Dog 3	0.76	0.33	0.98	0.76	0.69	0.34	0.88	0.83	**0.86**	0.18	0.95	0.40	**0.23**	**0.07**	0.25	**0.19**
**Gold**																
**Activity**	**Stand**				**Sit**				**Lie**				**Walk**			
	**Avg**	**Sd**	**Md**	**R**	**Avg**	**Sd**	**Md**	**R**	**Avg**	**Sd**	**Md**	**R**	**Avg**	**Sd**	**Md**	**R**
Dog 1	0.94	0.12	**1.00**	0.39	**0.96**	0.08	**1.00**	0.25	0.85	0.24	**1.00**	0.60	0.47	**0.29**	0.37	**0.91**
Dog 2	0.83	0.25	0.94	0.77	0.92	0.09	0.95	0.24	0.64	0.24	0.73	0.72	0.31	0.13	0.39	0.32
Dog 3	0.94	0.08	0.96	0.22	0.86	0.12	0.87	0.31	0.83	0.29	0.95	0.86	**0.18**	**0.03**	**0.17**	**0.08**

**Table 4:** The computed *p*-values of Mann-Whitney test (n = 27) regarding the electrodes in different activity modes.

	Stand	Sit	Lie	Walk
Peak/Polymer	**0.027**	**0.029**	0.510	0.117
Peak/Gold	**0.000**	**0.000**	0.897	0.418
Polymer/Gold	0.053	0.252	0.184	0.354
